# Thermostable Cellulases / Xylanases From Thermophilic and Hyperthermophilic Microorganisms: Current Perspective

**DOI:** 10.3389/fbioe.2021.794304

**Published:** 2021-12-15

**Authors:** Samaila Boyi Ajeje, Yun Hu, Guojie Song, Sunday Bulus Peter, Richmond Godwin Afful, Fubao Sun, Mohammad Ali Asadollahi, Hamid Amiri, Ali Abdulkhani, Haiyan Sun

**Affiliations:** ^1^ Key Laboratory of Industrial Biotechnology, Ministry of Education, School of Biotechnology, Jiangnan University, Wuxi, China; ^2^ Department of Biotechnology, Faculty of Biological Science and Technology, University of Isfahan, Isfahan, Iran; ^3^ Department of Wood and Paper Science and Technology, Faculty of Natural Resources, University of Tehran, Karaj, Iran; ^4^ Institute of Tropical Bioscience and Biotechnology, Chinese Academy of Tropical Agricultural Sciences, Haikou, China

**Keywords:** thermostable cellulase, thermostable xylanase, thermophilic microorganism, thermostability, lignocellulose, genetic engineering, enzyme hydrolysis

## Abstract

The bioconversion of lignocellulose into monosaccharides is critical for ensuring the continual manufacturing of biofuels and value-added bioproducts. Enzymatic degradation, which has a high yield, low energy consumption, and enhanced selectivity, could be the most efficient and environmentally friendly technique for converting complex lignocellulose polymers to fermentable monosaccharides, and it is expected to make cellulases and xylanases the most demanded industrial enzymes. The widespread nature of thermophilic microorganisms allows them to proliferate on a variety of substrates and release substantial quantities of cellulases and xylanases, which makes them a great source of thermostable enzymes. The most significant breakthrough of lignocellulolytic enzymes lies in lignocellulose-deconstruction by enzymatic depolymerization of holocellulose into simple monosaccharides. However, commercially valuable thermostable cellulases and xylanases are challenging to produce in high enough quantities. Thus, the present review aims at giving an overview of the most recent thermostable cellulases and xylanases isolated from thermophilic and hyperthermophilic microbes. The emphasis is on recent advancements in manufacturing these enzymes in other mesophilic host and enhancement of catalytic activity as well as thermostability of thermophilic cellulases and xylanases, using genetic engineering as a promising and efficient technology for its economic production. Additionally, the biotechnological applications of thermostable cellulases and xylanases of thermophiles were also discussed.

## Introduction

**FIGURE 1:**
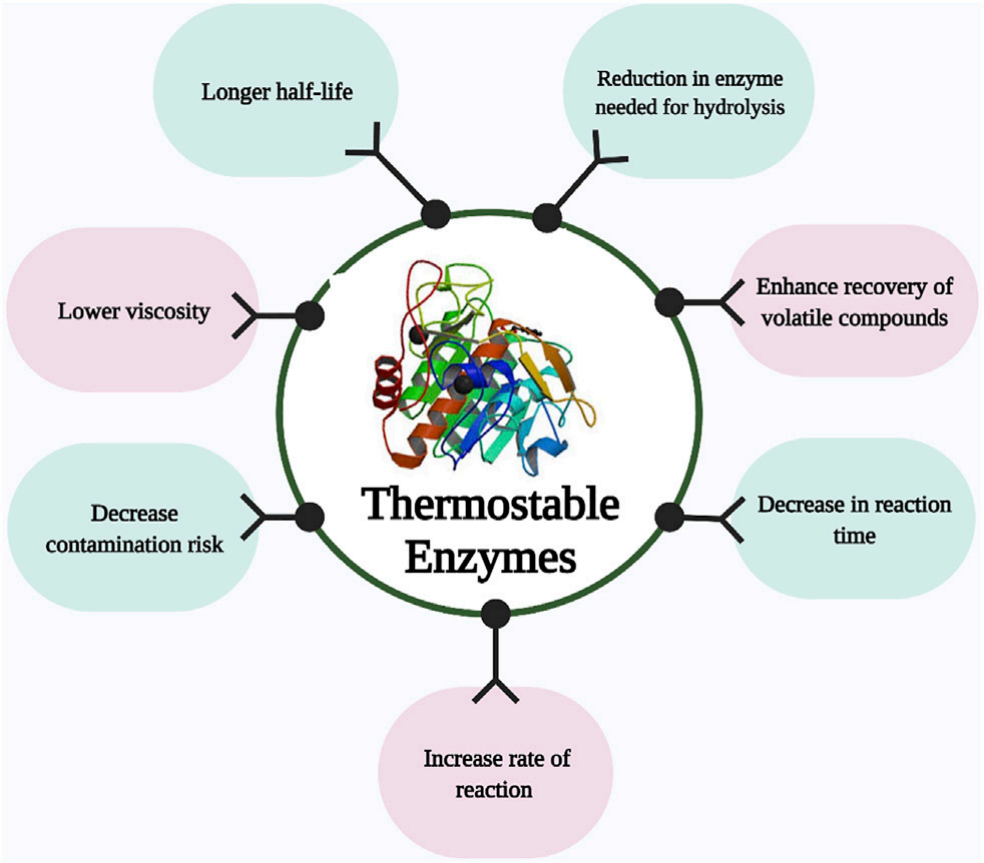
Advantages of enzymatic hydrolysis carried out at elevated temperature.

Enzymes are natural catalysts capable of accelerating highly efficient chemical reactions. However, the inability of these catalyst to withstand harsh industrial conditions have limit their application in various industries. After a sharp increase in biofuel-related research studies during 2004–2008, the enzymatic hydrolysis of cellulose and hemicellulose content of lignocellulose has been identified as one of the main challenging steps of the lignocellulose bioconversion, an important part of circular bioeconomy. Moving from mesophilic to thermophilic enzymes is a vital approach to address the technical and economical drawbacks of typical enzymatic hydrolysis gaining attention in recent years (Arora et al., 2015; Boyce and Walsh, 2018; Fusco et al., 2018b; Singh N. et al., 2021). Lignocellulosic biomass, as an inexpensive and abundant substrate, can be used for the manufacturing of second-generation biofuels as well as a variety of valuable chemicals through biorefinery platforms (Benedetti et al., 2019; Ho et al., 2019). Bioethanol produced from lignocellulosic biomass is an important source of renewable transport fuel since it can reduce greenhouse gas emissions, decrease fossil fuel dependency, improve energy security, and decrease food prices without compromising food production (Majidian et al., 2018; Tian et al., 2018; Patel et al., 2019; Solarte-Toro et al., 2019; Lee and Park, 2020). However, lignocellulosic biomass needs to be degraded prior to use by microorganisms because most of microorganisms cannot use it directly. There is numerous chemical, physical, and enzymatic processes for the degradation of lignocellulosic biomass (Nwamba et al., 2021a). Enzymatic methods offer many advantages over chemical and physical pretreatments as they are environmental friendly, and do not need harmful chemicals such as acids or bases as among all known processes, the enzyme-based hydrolysis of lignocellulose feedstock using cellulase and xylanase is best because of its enhanced specificity, toxic substance is not produced, and no substrate loss (Mohsin et al., 2019). Cellulase and xylanase are the main enzymes required for the hydrolysis of lignocellulosic biomass (Mukasekuru et al., 2020; Ariaeenejad et al., 2021). However, the enzymatic process has been hampered by the cost of enzymes (Pihlajaniemi et al., 2021). Despite decades of research on reducing the cost of enzymatic hydrolysis, its contribution to the economics of bioethanol production process, i.e., 0.68–1.47 $ per gallon of produced ethanol, is still too high to be feasible (Klein-Marcuschamer et al., 2012). Regardless of the tremendous research progress recorded in this research field, a more recent study shows the average cost of bioethanol production from lignocellulosic biomass ranges from $1.91 to $3.48 per gallon ethanol (Saini et al., 2020). It has been emphasized by different researchers like Stephanopoulos (Gregory, 2007) that it is very important to decrease the cost of enzyme down to 3–4 cents per gallon of ethanol before commercializing the lignocellulosic bioethanol. Increased enzyme activity at high temperature in the trade-off between activity and stability has been suggested as a possible approach to reduce the enzyme dosage and consequently the cost contribution of enzymes. It has been suggested that these limitations could be overcome by using highly thermostable enzymes from thermophilic and hyperthermophilic microbes (Viikari et al., 2007; Yeoman et al., 2010). Furthermore, saccharification carried out at an elevated temperature is very advantageous as shown in Figure 1. Concerning configurations of processes and processes with high specificities, thermostable enzymes have more stability, significantly reducing the quantity of enzymes needed for hydrolysis and decrease in reaction time (Yang et al., 2018; Karnaouri et al., 2019). At elevated temperatures, a decrease in viscosity enhances the diffusion rate of the substrate for effective enzymatic degradation (Bhalla et al., 2013). Thermostable enzymes increase the rate of reaction, enzymes tend to have a longer half-life, decrease contamination risk when compared to enzymes from mesophiles. In addition, they enhance the solubility of the feedstock, recoveries of volatile compounds, and also enzymatic efficiency is enhanced in industrial processes (Arora et al., 2015; Yadav et al., 2018). Before enzymatic degradation, lignocellulosic biomass usually undergoes acidic or alkaline pretreatment followed by neutralization. Thermo-acidophilic and thermo-alkalophilic enzymes could prevent the need for this neutralization step (Bhalla et al., 2013).

**GRAPHICAL ABSTRACT:**
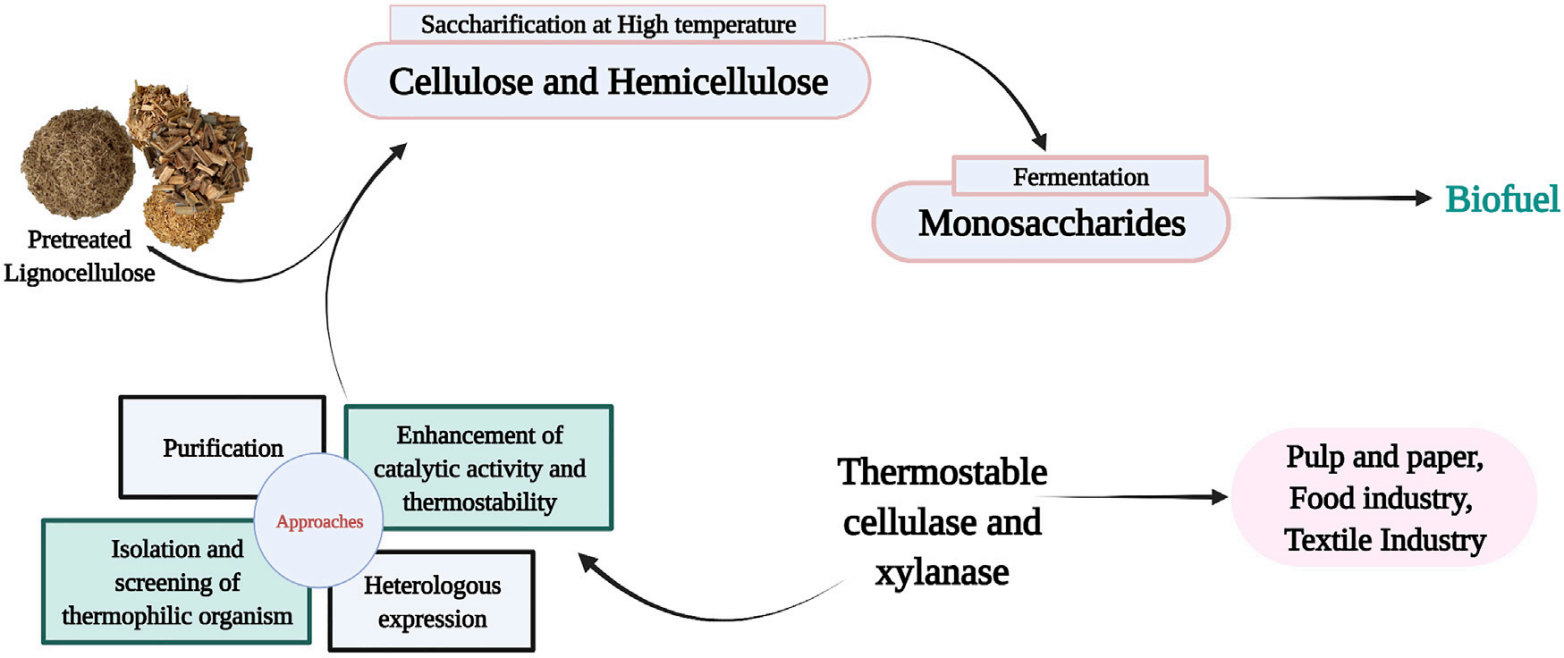


A deep understanding of thermophilic microorganisms is required to develop processes for commercializing thermostable or thermotolerant enzymes, especially cellulases and xylanases. In prokaryotes, thermophilic microorganisms include photosynthetic bacteria, blue-green algae, spore-forming bacteria (*Bacillus* and *Clostridium*), actinomycetes, bacteria that oxidize and reduce sulfur, bacteria that oxidize and produce methane, and Gram-negative aerobes (Bala and Singh, 2019a). The genera *Bacillus*, *Thermus,* and *Clostridium* contain microorganisms that can grow between the temperatures of 55°C and 70°C. While the temperature ranges for the growth of hyperthermophiles are between 65°C and 110°C, they are mainly archaea and bacteria, but because of their limited membrane adaptation at elevated temperatures and unfavorable growth and development conditions, eukaryotes are rarely found in this group (Boyce and Walsh, 2018; Straub et al., 2018; Bala and Singh, 2019b; Bala and Singh, 2019a). There are more than seven species, two genera, and order of bacteria belong to the hyperthermophiles. However, most of them are archaea since they excellently adapt to extreme temperatures in the environment (Bala and Singh, 2019a). Fungi are the only group in eukaryotes with the unique capability of thriving in high temperatures and are of special interest due to their potential to synthesize a remarkable range of heat-stable enzymes (Prasad, 2019). Thermophilic fungi can proliferate in many different natural environments, such as habitats and soil where the decay of dead plant materials occurs, compost piles, nests of birds, and municipal waste. Hundred years ago*, Mucor pusillus* was isolated from bread as the first thermophilic fungus. A thermophilic fungus known as *Thermomyces lanuginosus* has been discovered on potatoes inoculated with garden soil after several years (Bala and Singh, 2019a; Prasad, 2019).

Almost all known enzymes, e.g., proteases, lipases, amylases, cellulases, xylanases, and cell-associated enzymes (trehalase, invertase, and glycosidase), are obtainable from thermophilic and thermotolerant microorganisms (Bala and Singh, 2017). In contrast to mesophilic enzymes, enzymes secreted from thermophilic microorganisms are very active and extremely stable in the presence of alcoholic substances, detergents and organic solvents, thereby negating the usage of expensive industrial catalytic agents which are useful in different industries (Arora et al., 2015; Bala and Singh, 2019a; Prasad, 2019). A lack of information on thermostabilized enzymes with high specific activities is limited in the literature, while limited information is available on how to improve their production and thermostability from thermophilic organisms. Thus, the present review aims at giving an overview of the most recent thermostable cellulases and xylanases isolated from thermophilic and hyperthermophilic microbes and from metagenomes with emphasis placed on trends recently used to manufacture these enzymes in mesophilic host in large quantity and enhancement of specific activity as well as thermostability of thermophilic cellulases and xylanases, while also highlighting their biotechnological applications.

## Cellulase and Xylanase of Thermophiles

### Cellulase of Thermophiles

Cellulose hydrolysis is mainly carried out by β-endoglucanase (EC 3.2.1.4) as the polymer is cleaved to produce short oligosaccharides followed by the simultaneous action of 1,4 β-cellobiohydrolase (EC 3.2.1.91) and β-glucosidase (EC 3.2.1.21), which hydrolyzes it to glucose (Prasad et al., 2018). Cellulases have broad commercial applications because they convert lignocellulosic biomass into simple monosaccharides via enzymatic degradation, which can then be used to manufacture several valuable products in the industry (Patel et al., 2019). Furthermore, cellulase can be used for many other applications, including waste management, pigment extraction, and bioactive molecules extraction from plant materials (Ghosh et al., 2020). Thermostable cellulase is of great interest in the depolymerization of complex lignocellulose polymers. Chemically pretreated biomass can be hydrolyzed by this group of cellulolytic enzymes from thermophilic organisms (Azadian et al., 2017). These enzymes can be secreted as free enzymes or consolidated into complexes of multienzymes known as cellulosome, which can efficiently release sugar when directly applied to a cellulosic substrate (Thomas et al., 2014a; Singh N. et al., 2021).

#### Characteristics and Properties of Thermostable Cellulase From Thermophilic Bacteria

A lesser amount of attention is paid to cellulases of bacterial origin since the fungi are known to secrete this catalyst more efficiently. The production of cellulase is known to occur both in aerobic and anaerobic bacteria. There are striking similarities and differences between bacteria that can grow aerobically and anaerobically concerning their cellulase enzyme systems, cell mass yields, and yields of lignocellulose polymer hydrolysis (Kuhad et al., 2016).

Many thermophilic bacteria have produced thermostable cellulolytic enzymes. These include *Bacillus, Geobacillus, Caldibacillus, Acidothermus, Caldocellum, and Clostridium* (Ghosh et al., 2020). The Hyperthermophilic anaerobe *Caldicellulosiruptor bescii,* isolated from a Kamchatka hot spring, contained highly active cellulases. This bacterium depolymerized cellulose substrate without treatment with chemical, and this was because it produced a multi-modular and multi-functional enzyme known as CelA that was superior to commercially available cellulases (Singh N. et al., 2021). Recently, cellulolytic enzymes of *C. bescii* were used to build a “designer cellulosome” that showed stability and activity at 75°C (Kahn et al., 2019). A hyperthermophilic bacterium *Dictyoglomus turgidum* has a gene (Dtur_0,462) that encodes β-glucosidase was expressed in *E. coli*. This enzyme has a maximum activity at a temperature of 80°C and pH 5.4. This β-glucosidase is extremely stable over a pH range of 5–8, and it retains 70% of its relative activity after 2 h at 70°C. It proved to be suitable for the industrial production of bioethanol, because the enzyme showed high glucose and ethanol tolerance (Fusco et al., 2018a). A cellulolytic gene from *Thermotoga naphthophila* RKU-10T was isolated and expressed in *E. coli*. The purified TnCel12B shows maximum relative activity at pH 6.0 and temperature of 90 °C. After incubation at 85 °C, it maintained 100% activity after 8 h with excellent stability over a broad temperature range (50–85°C) and pH range (5.0–9.0) (Akram and Haq, 2020). A gene from hyperthermophilic archaea *Sulfolobus shibatae* that encodes endo-1,4-β-d-glucanase was cloned and overexpressed in *E. coli*. The recombinant enzyme has relative optimum activity at 95–100°C. The enzyme showed excellent resistance to high temperatures and 100% relative activity was detected after 60 min at 75°C, 80°C, and 85°C; 98, 90, and 84% of original relative activity were observed after 120 min at 75°C, 80°C, and 85°C, respectively (Boyce and Walsh, 2018). Likewise, there have also been some discoveries of new thermostable cellulases from archaea. Oil reservoir metagenome cellulase F1 proved to contain two cellulase modules, likely derived from two different archaeal cellulases. Compared to commercially available enzymes, the fusion enzyme proved to be more thermostable and active (Lewin et al., 2017). A summary of the thermostability of cellulase from thermophilic and hyperthermophilic bacteria is shown in Table 1.

**TABLE 1:** Thermostable cellulases from various thermophilic microorganism and their characteristics.

Microorganism	Enzyme	Optimum pH	Optimum temperature	Specific activity	Thermostability/half-life	References
**Geobacillus sp. T1**	Cellulase	6.5	70°C	ND	Stable for 1 h at 60°C	Assareh et al. (2012)
**Thermotoga naphthophila RKU-10** ^**T**^	endo-1,4-β-glucanase	6	90°C	1664 U mg^−1^	Half-life of 180 min at 95°C	Akram and Haq, (2020)
***Dictyoglomus turgidum***	β-glucosidase	5.4	80°C	160 U mg^−1^	After incubation at 70°C for 2 h, it retained 70% of its activity	Fusco et al. (2018a)
**Dictyoglomus thermophilum**	endo-1,4-β-glucanase	5.0	50–85°C	7.47 ± 0.06 U/mg	It retained 80% of its relative activity after incubation at 50°C for 135 days	Shi et al. (2013)
***Sulfolobus shibatae***	endo-1,4-β-glucanase	3–5	95–100°C	ND	Retained 98, 90, and 84% of its activity at 75°C, 80°C, and 85°C, respectively after 120 min	Boyce and Walsh, (2018)
**Bacillus licheniformis A5**	Cellulase	6.0	50°C	ND	Retained 82% of its activity after 120 min at 80°C	Yang et al. (2021)
**Aspergillus heteromorphic**	Cellulase	4.5	60°C	ND	Retained 60.0% of its activity after 1 h at 90°C	Singh et al. (2009)
**Sporothrix carnis**	Cellulase	5.0	80°C	ND	Retained 75% of its activity after 300 min at 80°C	Olajuyigbe and Ogunyewo, (2016)
**Paecilomyces thermophila**	β-glucosidase	6.0	65°C	ND		Yan et al. (2012)
**Putranjiva roxburghii (PRGH1)**	β-glucosidase	5.0	65°C	ND	75°C for 60 min	Kar et al. (2017)
***Thermoascus aurantiacus***	β-glucosidase	5.0	70°C	23.3 U/mg	It maintained 70% of its relative activity after incubation at 60°C for 1 h	Hong et al. (2007)
***Geobacillus* sp. HTA426**	Cellulase	7.0	60°C	ND	Stable at 50–70°C for 300 min	Potprommanee et al. (2017)
***Talaromyces emersonii***	β-glucosidase		71.5°C	482.8 U/mg	Half-life of 62 min at 65°C	Murray et al. (2004)
***Talaromyces emersonii***	Cellobio-hydrolase	5.0	68°C	ND	Half-life of 68 min at 80°C	Grassick et al. (2004)
***Talaromyces emersonii* CBS394.64**	endo-1,4-β-glucanase	4.5	90°C	ND	Highly thermostable at 70°C	Wang et al. (2014)

#### Characteristics and Properties of Thermostable Cellulase From Thermophilic Fungi

A variety of applications require thermostable enzymes; which thermophilic fungi provide. These thermostable enzymes, which are found in their habitat, have recently received significant attention, especially in biomass degradation (Thapa et al., 2020). Many thermophilic fungi have been identified to be able to produce highly thermostable cellulase, including Sporotrichum sp. (Ishihara et al., 1999), Thermoascus aurantiacus (Gomes et al., 2000), Talaromyces emersonii (Murray et al., 2001), and Syncephalastrum racemosum (Wonganu et al., 2008). They exhibit maximum activity at 70°C, 80°C, 75°C, and 70°C, respectively. Purified cellulases secreted by these eukaryotic microorganisms have undergone structurally and functionally characterization (Patel et al., 2019). A gene TeEgl5A that encodes a highly thermostable β-endoglucanase was isolated from a thermophilic fungi *Talaromyces emersonii* CBS394.64 and it was overexpressed in *Pichia pastoris.* After purification, the recombinant β-endoglucanase shows optimal relative activity at a temperature of 90°C and pH of 4.5. It has very high stability at 70°C and over a wide pH range of 1.0–10.0, and it is very resistant to the majority of metal ions, proteases, and sodium dodecyl sulfate. TeEgl5A possesses a wide range of substrate specificity and shows increased activity against polymers that contain β-1,4-glycosidic bonds and β-1,3-glycosidic bonds (Wang et al., 2014). *Thermoascus aurantiacus* possess the ability to secret a highly thermostable cellulase for biomass deconstruction (Mohsin et al., 2019). The best cellulase producer among fungi was thought to be Trichoderma sp. but it can be susceptibility to product inhibition (Akram et al., 2018).

Both novel thermostable β-glucosidases from filamentous fungi were expressed in *Trichoderma reesei.* They both have an optimum temperature at 60°C and pH 5.0 both enzymes were highly thermostable. Enzymes from CEL3a and CEL3b were incubated at pH 5.0; and 60°C they retained 98 and 88% of their relative activity after 6 h of incubation (Colabardini et al., 2016). The cellulase activity of Sporotrichum thermophile (Coutts and Smith, 1976) and Talaromyces emersonii (Folan and Coughlan, 1978) is almost the same as the cellulase activity of a mesophilic fungi Trichoderma reesei. Compared to the relative cellulase activity of Trichoderma viridae, some thermophilic fungi C. thermophile, S. thermophile, and T. aurantiacus were identified to produce cellulase that is twice or thrice greater in activity (Akram et al., 2018). There have been more significant temperature stable proteins in thermophilic bacteria and hyperthermophilic archaea than in thermophilic fungi (Patel et al., 2019). A summary of the thermostability of cellulase from thermophilic fungi is shown in Table 1.

### Xylanase of Thermophiles

Plants and algae contain hemicellulose, a complex polymeric carbohydrate found in their cell walls. About 33% of the world’s green organic carbon is made up of hemicellulose, which is composed primarily of xylan (Bhardwaj et al., 2019; Yang H. et al., 2020; Nwamba et al., 2021b; Shen et al., 2021; Sun et al., 2021). Hemicellulose is hydrolyzed by a variety of enzymes due to its heterogeneous structural composition (Zhuo et al., 2018). Specifically, endo-mannanases are responsible for cleaving of β-1,4 d-manno-pyranosyl bonds a linear and branched oligosaccharide is produced within the main chain (Nwamba et al., 2021b). Different types of microorganisms produce xylanases including thermophilic/thermotolerant fungi, yeast, and thermophilic/extreme thermophilic bacteria.

#### Characteristics and Properties of Thermostable Xylanases From Thermophilic and Hyperthermophilic Bacteria

Thermophilic and hyperthermophilic bacteria such as *Bacillus licheniformis* (Raj et al., 2018) *Rhodothermus marinus* (Karlsson et al., 2004), Caldicoprobacter algeriensis (Mhiri et al., 2020), *Thermococcus zilligii*, *Sulfolobus solfataricus,* and *Pyrodictium abyssi* have been extensively studied to produce highly thermostable xylanase. The majority of the xylanase produced from these thermophiles were identified to be members of families 10 and 11 of glycoside hydrolases (GH) (Ghosh et al., 2020). *Geobacillus* sp. Strain WSUCF1 has drawn attention lately because it produces highly thermotolerant xylanase with an exceptional thermostability with half-lives of 18 days at 60 °C and 12 days at 70°C (Bhalla et al., 2015). In another study, a gene that encodes GH10 endo-xylanase was isolated from Geobacillus sp. WSUCF1 and overexpressed in *E. coli*. After purifying the endo-xylanase it was tested against birchwood and showed maximum relative activity at a temperature of 70°C and pH 6.5. It was discovered to be highly thermostable at 60°C retaining 50% and also at 50°C retaining 82% of its original activity after 60 h (Bhalla et al., 2014). Similarly, a gene for xylanase xynBCA encoding a polypeptide of 439 residues (XynBCA) was isolated from Caldicoprobacter algeriensis and overexpressed heterologously in *E. coli* BL21 (DE3). The purified thermostable xylanase was optimally active at pH 6.5 and 80°C. It exhibited excellent thermostability with a half-life of 20 min at 80°C (Mhiri et al., 2020). A xylanase from *B. licheniformis* had optimum relative activity at pH 9.0 and 60°C and maintained 80% of its relative activity when incubated for 1 h at a 60°C (Raj et al., 2018). Some thermostable xylanases have also been reported from thermohalophiles, thermoacidophiles, and thermoalkaliphiles. For example, thermostable xylanase from thermoacidophilic Alicyclobacillus sp. was overexpressed heterologously in *E. coli* and was reported to have a broad pH range (3.8–9.4) and retained 90% of its relative activity when incubated for 1h at 60°C (Bai et al., 2010). Thermostable xylanase from halophilic bacterium, Thermoanaerobacterium saccharolyticum NTOU1, has a very high resistance to high salt concentration (when incubated in 2 M NaCl for 24 h it maintained 71% of its relative activity). Also, 50% of the relative activity of this xylanase was maintained when incubated at 65°C for 0.91 h (Hung et al., 2011). Similarly, thermo-alkalophilic xylanase was cloned from *Enterobacter* sp. MTCC 5112 and maintained 90% of its relative activity when incubated at pH 9.0 and 80°C for 0.66 h. It also maintained 85 and 64% of its relative activity at 60°C and 70°C after 18 h, respectively (Khandeparkar and Bhosle, 2006). Table 2 demonstrates a summary of thermostable xylanases from thermophilic and hyperthermophilic bacteria.

**TABLE 2:** Thermostable xylanases from various thermophilic microorganism.

Microorganism	Enzyme	Optimum pH	Optimum temperature	Specific activity	Thermostability/half-life	References
**Caldicoprobacter algeriensis**	xynBCA	6.5	80°C	117 U/mg	20 min at 80°C	Mhiri et al. (2020)
***Streptomyces griseorubens* LH-3**	Endo-xylanase	5.0	60°C	767.2 U/mg	Retained 60% of its activity at 50°C for 1 h	Wu et al. (2018)
**Chaetomium sp. CQ31**	Xylanase	6.5	85°C	2489 U/mg	It maintained over 90% of its relative activity at 60°C for half an hour	Yu et al. (2021)
**Thermotoga maritima, TmxB**	Endo-β-1,4-xylanase	5.0	100°C	ND		Yang et al. (2020b)
**Thermotoga neapolitana**	hemicellulytic	6.0	90°C	ND		Benedetti et al. (2019)
**Dictyoglomus turgidum**	Endomannase	5.4	70°C	ND	It retained 50% of its relative activity when incubated for 2 h at 70°C	Fusco et al. (2018b)
**Dictyoglomus turgidum**	β-xylosidase	5.0	98°C	ND	retained over 90% of its relative activity within this range of temperature 80°C–95°C	Tong et al. (2021)
**Dictyoglomus turgidum**	β-xylosidase	5.0	75°C	6.79 U/mg	retaining 88% activity at 65°C for 60 min	Li et al. (2020b)
**Acinetobacter Johnsonii**	Xylanase	6.0	55°C	ND	retained 80% relative activity after 60 min at 65°C	Xue et al. (2019)
**Thermoascus aurantiacus M-2**	Xylanase	5.0	75°C	ND	Highly Stable for 2 h from 30°C to 80°C temperature range	Ping et al. (2018)

#### Characteristics and Properties of Thermostable Xylanases From Thermophilic Fungi

Different thermophilic fungi such as *Thielavia terrestris* (García-Huante et al., 2017)*, Rhizomucor pusillus* (Hüttner et al., 2018)*,* and *Corynascus thermophiles* (van den Brink et al., 2013) have been reported as very good sources of thermostable xylanase. It was recently shown that *Thielavia terrestris* Co3Bag1 produces a highly thermostable xylanase with a molecular weight of 82 kDa. This enzyme has maximum relative activity at pH 5.5 and 85°C. After incubation at 65°C, it has a half-life of 23.1 days (García-Huante et al., 2017). It was found that R. pusillus isolated from maize silage could produce 824 (U/g) of xylanase that is highly stable at 75°C (Robledo et al., 2016). Genes encoding putative xylan degrading enzymes were identified in the thermophilic fungus R. pusillus (Hüttner et al., 2018). A thermophilic xylanase (XynC01) was identified in the thermophilic fungus Achaetomium sp. Xz-8 and overexpressed in P. pastoris. It exhibited maximum relative activity at pH 5.5 and 75°C with very good stability over a broad range of pH (pH 4.0–10.0) and at temperatures of 55°C and lower. This thermostable xylanase exhibits excellent tolerance to metal ions and chemical reagents. Combining this xylanase with commercial β-glucanase enhanced its performance (38.50%). These properties make it suitable for industrial applications particularly in breweries (Zhao et al., 2013). Another thermostable xylanase (Xyn11A) was obtained from Corynascus thermophilus and it expressed in P. pastoris. This thermostable xylanase has maximum relative activity at pH 7.4 and 70°C. When incubated at 50°C and 60°C for 60 min, Xyn11A maintained more than 90% of its relative activity. Xyn11A shows great stability over a wide pH range (2.0–11.0). Its relative activity was not inhibited by metal ions, making it suitable candidate for industrial applications (Yang and Zhang, 2017). In another study, a gene encoding a highly thermostable xylanase in *Paecilomyces thermophile* was overexpressed in *P. pastoris.* XynA shows maximum activity at 75°C and is highly stable when exposed to 80°C for 30 min. XynA produces xylobiose and xylotriose as its main products after hydrolyzing birchwood xylan, beechwood xylan and xylooligosaccharides (Fan et al., 2012). The thermostable fungus, Thermoascus aurantiacus M-2 produced xylanase with relative molecular mass of approximately 31.0 kDa showing optimal relative activity at 75°C and pH 5.0. It was active over a wide range of pH (pH 2.0–10.0) and it showed excellent stability over a wide temperature range (30°C–80°C) for 120 min. The xylanase relative activity was enhanced by Mn^2+^ and Ag^+^ to 120.0 and 119.6%, respectively (Ping et al., 2018). Table 2 demonstrates a summary of thermostable xylanases from thermophilic fungi.

### Bioprospecting Novel Thermostable and Hyper-Thermostable Cellulase and Xylanase From Metagenomes

Increasing interest has been shown in finding thermostable cellulases and xylanases which lie in huge unculturable microbial diversity found in extreme habitats (Zhong et al., 2021). Metagenomics are widely used as very powerful techniques to identify, Isolate, and characterized novel thermostable enzymes with efficient catalytic activities from extreme environment which includes various environment that are usually characterized by extreme temperature like hot springs, deserts, compost, hydrocarbon reservoirs, hydrothermal vents, e. t.c. (Zarafeta et al., 2016; Madhavan et al., 2017b; Hebal et al., 2021). The ability of a microbes to survive in these extreme habitat makes their protein to be highly thermostable. Mining of novel biocatalyst through metagenomics can be carried out using functional-based screening of the expression libraries in which metagenomics expression libraries are constructed and screened for target enzyme activity. It can also be carried out through sequenced-based gene searches which rely on known conserved sequences, where target genes are amplified from metagenomic DNA using conserved sequences as primers and subsequently cloned into the appropriate expression systems (Li et al., 2009). The first attempt at discovering novel enzymes using a functional metagenomic approach was made by (Healy et al., 1995) from “zoolibraries” by cloning cellulases with temperature (60–65°C) and pH optima (6–7) Since then, metagenomics approaches have been used to screen and isolate thermostable cellulase from different ecological niches. A novel β-glucosidase like gene was cloned using function-based screening of metagenomic library from uncultured soil microorganism (Jiang et al., 2009). Similarly, a novel cellulase cocktail was screened and isolated from the camel rumen metagenome. This cellulase cocktail shows enhanced activity at high temperatures above 50°C when compared to the activity of the individual cellulase (Maleki et al., 2020). Another thermostable cellulase was also isolated and expressed from buffalo rumen metagenomic library, which was optimally active at temperature of 50°C and pH 5.0 (Pabbathi et al., 2021).

A novel xylanase was isolated from chicken cecum metagenome, which was highly active at high concentration of salt. It has high potential of application in chicken feed (Al-Darkazali et al., 2017). Furthermore, it was proposed that the primary composition of poultry feed is a high ratio of non-starch polysaccharides that include xylans and arabinoxylans, so the microorganisms capable of degrading these polysaccharides should be abundant in chicken intestine. A novel alkali-stable and thermostable GH11 endoxylanase encoding gene (Mxyl) was retrieved by functional screening of a compost soil metagenome. The recombinant xylanase (1,077 bp) shows optimum activity at 80°C and pH 9.0 (Verma et al., 2013). In another study, a xylanase gene was isolated and expressed in B. Megaterium from metagenomic DNA of cow dung*.* The recombinant xylanase was found to be optimally active at pH 7 and 75°C (Sun et al., 2015).

## Expression of Thermostable Cellulase and Xylanase

To conquer the current bottleneck of the synthesis of thermostable lignocellulolytic enzymes in large quantities, we must first figure out how to get them at a cost-effective price. Enzymes are routinely over-expressed with the help of recombinant DNA technology (Juturu and Wu, 2014). In nature, cellulases and xylanase are secreted by broad species of microorganisms. One of the best ways of obtaining new thermoenzymes is through the screening and isolation of cellulase and xylanase-producing microorganisms from nature (Parveen et al., 2019). Saprophytic microorganisms are typically responsible for secreting cellulases and xylanases from dead decaying organic matters. Several plant pathogens also secrete cellulases and xylanase (Wang L. et al., 2020). Microbes producing xylanase and cellulase are typically isolated from soil samples obtained from forest and nature preserves, hot springs, compost, sewage, animal manure, and bovine rumens. These enzymes are excreted outside the cell and have high quality, making cellulases and xylanases from fungi beneficial for industry (Cai et al., 2018; Joseph et al., 2019; Bhatia et al., 2021).

### In Bacteria

Recombinant protein expression has been used to increase productivity in a shorter duration and also reduce production costs. Homologous and heterologous expressions of cellulase and xylanase in bacteria, yeasts, filamentous fungi, and plants have been reported for increasing thermostable enzyme production (Juturu and Wu, 2012). The most regularly used bacteria used for the expression of recombinant proteins are *E. coli* and *Bacillus* (Juturu and Wu, 2014). Several factors contribute to the success of this host as a platform for recombinant expression of the protein, including rapid growth on cheap media, simplicity of the transformation procedure, and ease of isolation and purification of expressed proteins. However, the absence of repetitive codons and the need for specific post-translation modifications such as the formation of disulfide bond and glycosylation limit the ability to efficiently express xylanase heterologous in *E. coli* (Juturu and Wu, 2012). Most xylanases require N-glycosylation, whereas *E. coli* can only achieve simple O-glycosylation. However, an exception was observed in a glycosylated -xylosidase gene isolated from a thermophilic fungus, *P. thermophila*, and overexpressed in *E. coli,* yielding a titer of up to 98.0 U/ml (Juturu and Wu, 2012). Similarly, a gene that encodes a GH10 endo-xylanase was cloned from Geobacillus sp. WSUCF1 and heterologously expressed in *E. coli*. This recombinant protein showed a very high specific activity (461.0 U/mg), very high hydrolytic activity (92%), and a very high thermostability. Potential applications of this enzyme include pulp bleaching and biofuel production (Damm et al., 2016). This means glycosylation did not play a very vital role in keeping the activity of these enzymes. The expression of Endo-xylanase in *Lactobacillus* and *B. subtilis* is usually higher compared to *E. coli* because they are Gram-positive and they perform N-glycosylation (Upreti et al., 2003). The advantages of using *B. subtilis* heterologous expression of recombinant protein include lack of pathogenicity and endotoxins, no appreciable codon usage bias, and inexpensive protein purification methods (Wang et al., 2019). *Streptomyces* lividans is also a Gram-positive bacteria found in the soil, that has been utilized for the synthesis of the recombinant enzyme. it can also be used for the synthesis of secondary metabolites (Binnie et al., 1997). The ability of *Streptomyces* to secret high concentration of recombinant protein and the ease for bacterial transformation makes it a suitable host for enzyme expression. Furthermore, it has a proven track record for expressing enzymes that have been used for the synthesis of pharmaceutical drugs (Anné et al., 2012; Kang and Kim, 2021).

*Zymomonas mobilis* is an ethanol-tolerant bacterium that ferments a wide variety of monosaccharides and produces bioethanol in significant quantities (Kurumbang et al., 2019). It possesses some unique properties that make it an excellent candidate to be considered as a substitute for yeast in the manufacturing of bioethanol (Li et al., 2019). Utilizing this bacterium as an expression platform for recombinant protein shows about 12–30 times higher protein expression when compared to *E. coli* (Kurumbang et al., 2019). Other advantages include: relatively simple gene transformation techniques, and the ability to express recombinant protein both intracellularly and extracellularly. Expression plasmids can be kept either as autonomous replication DNAs or integrated into the host genome (Ray and Behera, 2017).

### In Yeast

*Saccharomyces cerevisiae* was the first yeast that was implemented for recombinant protein production in 1981 (Hitzeman et al., 1981). *Saccharomyces cerevisiae*, Pichia pastoris, Hansenula polymorpha, Kluyveromyces lactis, and Yarrowia lipolytica are the most common yeasts utilized for recombinant protein production. Their potential in food production is attributed to the non-production of toxins (Nøhr et al., 2003; Liu et al., 2012).

*S. cerevisiae* is a well-characterized eukaryotic microorganism that has been utilized for the synthesis of thermostable recombinant protein (Baptista et al., 2021). A larger number of cellulase and xylanase from thermophilic microorganisms have been efficiently expressed in *Saccharomyces cerevisiae* (Li J. et al., 2020).

*K. lactis* has so many advantages as a platform for the expression of a recombinant protein, which include easy genetic manipulation, application of both integrative and episomal expression vectors, it is known to produce a protein that is very useful in the food and dairy industry (Spohner et al., 2016). A thermostable endoglucanase was clone from Aspergillus fumigatus DBINU-1 and it was heterologously express in *K. lactis* this recombinant enzyme has optimal activity at pH 5.0 and 60°C (Rungrattanakasin et al., 2018). Similarly, an alkali-thermostable xylanase was cloned from *Bacillus* pumilus and it was expressed in *K. lactis* this shows very good stability at pH 12.0 retaining 74% of it Activity after incubation for 2 h it is suitable for application in pulp and paper industry (Thomas et al., 2014b).

*P. pastoris* is a methylotrophic organism and it was first used as a platform for heterologous expression in 1985 (Cregg et al., 1985). It is presently considered one of the most efficient systems for producing and expressing recombinant proteins (Potvin et al., 2012). *P. pastoris* can produce structurally and functionally stable recombinant proteins, particularly when the recombinant protein is derived from eukaryotic sources. It is capable of performing post-translational modifications, forming disulfide bonds, and folding proteins properly, and is a proliferator of essential proteases (Çalık et al., 2015). Its promising characteristics are leading to a range of interesting studies covering several aspects of improving the efficiency of the bioprocess, from strain engineering to bioprocess engineering (García-Ortega et al., 2019). A study was carried out recently on thermostable endoglucanase that was cloned from a Sclerotinia sclerotiorum and it was expressed in *P. pastoris* this recombinant enzyme was relatively stable at high temperature and it shows maximum activity at pH 7.0 and 60°C when compared to the native enzyme which have an optimum activity at pH 5.0 and 50°C (Chahed et al., 2018). Another study was carried out on high level expression of highly thermostable xylanase cloned from Chaetomium sp. CQ31 and overexpressed in *P. pastoris*. This novel recombinant xylanase exhibit high specific activity against oat–spelt xylan (2, 489 U/mg), beechwood xylan (1522 U/mg), birchwood xylan (1067 U/mg), and arabinoxylan (1208 U/mg), with an optimum activity at pH 6.5 and 85°C. This xylanase have a great potential of application in the brewery industry (Yu et al., 2021).

### In Filamentous Fungi

Many filamentous fungi are excellent producers of extracellular protein, and their ability to secrete a wide array of proteins and to facilitate the post-translational modification of proteins makes them more advantageous than bacteria for heterologous metabolite production (Khare et al., 2020). It has been reported that *A. niger* can produce 25–30 g/L of glucoamylase, and *Trichoderma species* can produce 100 g/L of extracellular proteins, proving the importance of protein production and secretion for these organisms. Recombinant DNA technology offers an easy way to express heterologous cellulases and xylanases in fungi due to their high protein secretion ability (Ward, 2012). Gene fusion strategies, overexpression of chaperones, or selecting host strains deficient in protease may be used to attain higher titer of recombinant proteins from filamentous fungi (Juturu and Wu, 2014). Cellobiohydrolase gene was cloned from *Trichoderma virens* cDNA and expressed in *A. niger*. This thermostable enzyme attained optimum activity at pH of 4.0 and 60°C. This partially purified enzyme shows a very high inhibition constant (Ki) compared to other fungal cellobiohydrolases (Wahab et al., 2019). Similarly, a highly thermostable xylanase B gene was cloned from hyperthermophilic bacteria Thermotoga maritima and it was heterologously express in *A. niger*. This thermostable xylanase exhibit optimum activity at very high temperature >90°C (Zhang et al., 2008).

### In Plant

Cloning of thermophilic enzymes in mesophilic species or production of the enzymes directly in plants is a possible method of thermophilic enzyme expression. Transgenic plants could be used to synthesize enzymes for the conversion of complex lignocellulosic polymers (Zhang et al., 2012). As a result of the expression of recombinant proteins in plants, enzyme production costs can be reduced, improving plant autohydrolysis and a very small quantity of exogenous enzyme loading will be needed during lignocellulosic biomass degradation (Phitsuwan et al., 2013). It is more expensive to produce enzymes in submerged fermentation reactions than it is to produce them in plants, as plants are easier to handle and can produce large volumes and high yields of protein (Wilson, 2009). Many studies have been done on the heterologous expression of thermophilic enzymes in plant biomass. A highly thermophilic endoglucanase gene (SSO1354) was cloned from *Sulfolobus solfataricus* and was functionally expressed in tobacco plant (Nicotiana tabacum). The recombinant enzyme (SSO1354) shows similar activity when compared to the wild type. The enzyme remains inactive at normal growth conditions, but it exhibited biological activity at elevated temperatures. There have been no adverse effects noticed during the development and growth of plants, and in ionic liquids used for pre-treatment of biomass, its heat-induced activation remains active (Klose et al., 2012). Heterologous expression of thermostable cellulytic and xylanolytic enzymes has been successfully carried out in plants which were very active under harsh conditions, which give way to combined pretreatment and enzymatic hydrolysis which is an effectual process of deconstruction of lignocellulose biomass into fermentable sugars. Similarly, another study was carried out on another hyper thermostable endoglucanase (E1) gene cloned from Acidothermus cellulolyticus and it was expressed in rice plant (*Oryza sativa*) under the optimized condition of Gt1 promoter. Interestingly, the enzyme produced in seeds shows similar properties to the endoglucanase in the native host (Zhang et al., 2012). Another endoglucanase gene has been cloned from *Acidothermus cellulolyticus* and heterologously expressed in different types of plants such as maize (Brunecky et al., 2011), rice (Oraby et al., 2007), potatoes (Dai et al., 2000), tobacco (Ziegelhoffer et al., 2009) and they have been considered as a good alternative for the production of biofuel in the nearest future.

## Strategies to Enhance Thermostability and Specific Activity of Cellulase and Xylanase of Thermophiles

**FIGURE 2:**
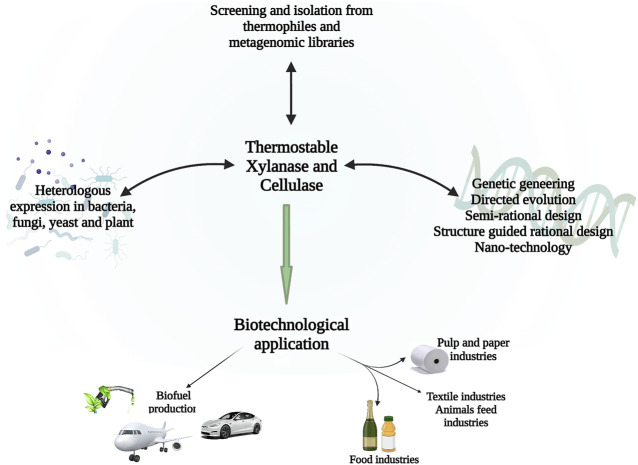
Schematic diagram showing a recent approach to generating enhanced cellulases and xylanases from thermophiles and the biotechnological application of these enzymes.

The applicability of thermostable cellulase and xylanase mainly depends on their productivity, thermostability, specific activity, a wide range of pH, and broad specificity of substrate (Behera and Ray, 2016). As shown in Figure 2. To increase the large-scale application of thermostable enzymes, different approaches have been utilized to enhance the thermostability of cellulase and xylanase from thermophiles and these include genetic modifications, expression regulation, and enzyme immobilization. (Boonyapakron et al., 2017; Zhang et al., 2017). The sequence alignment of the amino acid, crystallographic, and mutagenesis studies indicate that several minor modifications contribute to the enhanced temperature stability of xylanases. There is hydrogen bonds and ion pairs, as well as disulfide network formation, specifically in the N- to C-terminals and α-helix, of thermophiles and thermophilic glycoside hydrolases, and aromatic residues which, as a result, lead to the formation of ‘sticky patches’ on the surface of the protein (Ghosh et al., 2020).

### Genetic Engineering

#### Directed Evolution

Directed evolution is a way of mimicking natural evolution in the lab, in an accelerated fashion, and it can be used in the modification of proteins at will, to endow them with more desirable properties (Gargiulo and Soumillion, 2021). The application of directed evolution by random mutation has become a leading research field in protein engineering, particularly when there is insufficient information about crystal structures of the targeted proteins, Biocatalysts with greater power and efficiency can be generated through directed evolution, which is a widely used strategy in protein engineering (Goldsmith and Tawfik, 2012; Xu et al., 2016; Madhavan et al., 2017a). Direct evolution was utilized to enhance The thermostability and pH stability of *endo*-β-1,4-glucanase III which was active at 50°C for 30 min and pH range of 4.4–8. It was obtained from T. *reesei* QM9414 (Nakazawa et al., 2009). Another study was conducted recently on the enhancement of the temperature tolerance of the GH11 family using directed evolution, the variant Xyn376 showed excellent thermostability, it has a half-life at 70 °C for 410 min, which is 820-fold greater after comparing it with the wild-type enzyme (Xing et al., 2021). Similarly, the specific activity of *Bacillus stearothermophilus* xylanase A (BaxA) was increased by 3.5-fold (9.38 Umg^−1^) using directed evolution (Xu et al., 2016). The cellulase activity of a recombinant enzyme from *Thermotoga neapolitana* was enhanced using Random mutagenesis, the best mutants obtained when compared to the wild type shows 2.7-, 5- and 4.8- fold increase in activity against CMC, RAC and Avicel respectively (Basit et al., 2019). The use of mutations is an accepted method to improve recombinant proteins at a genetic level, although screening hundreds or thousands of mutants is tedious (Stephens et al., 2014).

#### Semi-Rational Design

Bioinformatics analysis is used to provide conserved regions of thermostable enzymes or active sites for mutation through semi-rational design (Wang et al., 2018; Wang H. et al., 2020). A semi-rational design was done to alter the activity of a chitinase PpChi1 cloned from *Paenibacillus pasadenensis* CS0611. This mutant S244C-I319C/T259P with proline substitution and disulfide bond introduction exhibits increased specific activity at elevated temperatures as well as a 26.3-fold increase in half-life value at 50°C. It also shows an increase in half-inactivation temperature with a 7.9°C when compared to the wild type protein (Xu et al., 2020). A single point mutation was introduced into *Penicillium canescens* xylanase PcXylA, resulting in a 2.5-fold higher half-life at 50–60°C (Denisenko et al., 2017). In recent years, site-directed mutagenesis and directed evolution have attracted much attention. With the use of these genetic modifications, the best mutant xylanase FC06T was obtained, which has a higher maximum temperature (shift from 77°C to 87°C) and a very high catalytic efficiency (up to 90%) (Zhang et al., 2010).

#### Structure-Guided Rational Design

In contrast to random mutagenesis, rational design requires *in silico* simulation and substantial crystal structure information (De Simone et al., 2015). By generating purpose-specific sequences with protein structure data, SCHEMA minimizes structure disruption in chimeric proteins when they are recombined (Heinzelman et al., 2013). A study was carried out on two cellulases, each from *Bacillus* and *Geobacillus*, to show the mechanism of their thermostability by comparing the enzymes from the two bacteria. Biofuels and animal feed industries may find these cellulases useful because of their unique thermostability. It was discovered that the cellulase from *Bacillus* BsCel5A is less thermostable compared to the cellulase from thermophilic Geobacillus sp. 70PC53. Therefore, these two cellulases are ideal for studying the mechanisms that enable them to remain active at elevated temperatures (Chang et al., 2016). A new recombinant enzyme with excellent enhanced activity could be synthesized by combining crystal structure determinations and structure-guided SCHEMA recombination. (Patel et al., 2019). Disulfide bonds in enzymes play a very important role in their biotechnological application. Replacement of some amino acids that are not consistent with catalytic activity can inherently affect the enzyme structure by strongly affecting the rate of irreversible protein inactivation (Mrudula Vasudevan et al., 2019). The introduction of disulfide bride Q1C-Q24C at the N-terminal region of GH11 xylanase that was obtained from *Thermomyces lanuginosus* has led to the enhancement of melting temperature by 10°C (Wang et al., 2012) Between the N-terminal and -helix of Xyn2 and the β-sheet core two disulfide bonds were also introduced, which led to enhanced resistance to alkali and acid (Tang et al., 2017). Xyn12.2 was isolated from the termite gut symbiont metagenome its k_cat_ and catalytic activity was improved by an increase in disulfide bonds, and its thermostability and alkali stability also improved (Boonyapakron et al., 2017). Chemical covalent modification of enzymes can effectively improve and facilitate catalysis. This rational design, guided by the structures of tiny particles incorporated into selected reto covalently modified enzyme BtGH84 cloned from *Bacteroides thetaiotaomicron*, resulted in a 35-fold increase in relative enzyme activity (Darby et al., 2017).

#### Immobilization on Nano-Strcutures

Nanotechnology has given rise to new opportunities in different biotechnological field, particularly in enzymes field which have been used in the enhancement of enzyme catalytic activity, thermostability, reusability and many other characteristics through enzyme immobilization (Rai et al., 2019; Gkantzou et al., 2021). The movement of enzymes at elevated temperature is limited by immobilization, which leads to enhanced stability of the enzyme. Furthermore, thermostable enzyme offers greater reaction rate, lower diffusional limitations and enhanced yields (Lee and Au-Duong, 2018). The separation of enzymes is made easier with magnetic nanoparticles, which allows them to be reused, and they also reduce processing costs (Ingle et al., 2017). Recently a research was carried out on β-glucosidase obtained from *Thermotoga maritima* (Tm-β-Glu) and it was functionally immobilized on magnetic nanoparticles (MNPs) with chitin (Ch), this research shows that the novel thermostable chitin-binding domain had the highest binding capacity and Galacto-oligosaccharide synthesis when it was compared with native production of enzyme. In addition, magnetic separation technology have been successfully used in recycling the immobilized β-glucosidase for repetitive batch-wise Galacto-oligosaccharide without significant reduction or loss of enzyme activity (Alnadari et al., 2020). Similarly, another investigation was carried out on thermostable β-glucosidase (Tpebgl3) of *Thermotoga petrophila* it was immobilized on macro-porous resin modified with polyethyleneimine and glutaraldehyde. The thermostability, pH stability and glucose tolerance were greatly enhanced, so also the relative activity of this enzyme was found to be 21% higher than that of free enzyme at 85°C (Shi et al., 2018). The thermal stability of free and immobilized β-galactosidase on amino- and cyanuric chloride-modified silica NPs were tested, and the investigation shows that immobilized enzymes retained 72% while the free enzyme only retained 35% of its initial activity after incubation at 60°C for 12 h (Banjanac et al., 2016).

## Biotechnological Application of Thermostable Cellulases and Xylanases

### Biofuel Production

A global crude oil problem hit in the 1970s, which prompted many industries to focus on using cellulases and xylanases in producing biofuel. Bioethanol, bio-butanol, acetoin, and 2,3-butanediol are produced by degrading cellulose and hemicellulose by cellulase and xylanase (Gusakov, 2013). As of today, ethanol production is not only needed because of oil crisis reasons, but it also reduces greenhouse gas emissions drastically (Bala and Singh, 2019b). This makes bioethanol the most common and widely used renewable fuel today (Pereira et al., 2016). Bioethanol derived from lignocellulose is environmentally friendly, and this process involves pretreatment, enzymatic degradation of polysaccharides into fermentable monosaccharides, and then fermentation of these sugars to bioethanol (Akram et al., 2018). Enzymatic saccharification of biomass is considered a vital step that contributes so much to the overall production cost, and this process can be enhanced by using thermostable enzymes produced from thermophilic microorganisms (Kuhad et al., 2016). Thermostability is a vital and desirable property needed to speed up the saccharification process of biomass (Candido et al., 2019). Within consolidated bioprocesses (CBP), genetically engineered microorganisms can perform enzyme production, enzymatic hydrolysis, and fermentation simultaneously (Antonov et al., 2017). Cellulolytic organisms can either be genetically modified to produce ethanol (CBP-I), or ethanol-producing organisms can be genetically modified to produce cellulases (CBP-II) (Houfani et al., 2020). Most consolidated bioprocesses (CBP) prefers thermophilic microorganisms over mesophilic microorganisms because thermophiles produce a catalyst that can facilitate the conversion of biomass to biofuel at elevated temperatures (Jiang et al., 2017). According to a study, *Trichoderma* has low β-glucosidase activity, which makes cellulose hydrolysis inefficient (Mohanram et al., 2013). Some thermophilic fungi, for example, *Sporotrichum thermophile* (Kaur et al., 2004), *Thermoascus aurantiacus* (Bala and Singh, 2017), *Scytalidium thermophillum*, and *Thielavia terrestris* can be used to replace it because they effectively degrade lignocellulosic biomass through the production of secretory enzymes (Berka et al., 2011). Therefore, these microorganisms have been proposed as an excellent candidate for the degradation of lignocellulosic polymers to simple fermentable monosaccharides that can be used in bio-ethanol industries (Berka et al., 2011). Molecular biology and metabolic engineering have enabled the introduction of strong promoters and regulatory elements to improve the expression of cellulase and xylanase (Beier et al., 2020). *T. reesei* cellulose yield and its activity level increased exponentially after overexpression of gene Cbh2, with a maximum yield of 119.49 IU L^−1^ h^−1^ (Li et al., 2017). A versatile cellulase system was constructed with a combination of genetic manipulations for the enzymatic saccharification of complex polymers and the production of powerful cellulase inducers (Gao et al., 2017).

Biobutanol production by reviving the old process using acetone-butanol-ethanol (ABE) fermentation has been suggested to address some important weak points of bioethanol, especially based on higher energy density of butanol and its compatibility with current fueling and engine infrastructures. Despite the great potential of butanol as a liquid fuel, its production process faced with important economic drawbacks. In this regard, efficient utilization of lignocellulosic wastes has been suggested based on the significant contribution of the carbon source in the economics of biobutanol production (Amiri et al., 2014). The typical process of biobutanol production from lignocellulose consists of 1) pretreatment, 2) enzymatic hydrolysis of cellulose, and 3) ABE fermentation of hydrolysate by solvent producing *Clostridia* Especially *Clostridium acetobutylicum* or *C. beijerinckii* to acetone, butanol, ethanol, acetic acid, and butyric acid (Amiri and Karimi, 2018). In this process, between 120 and 250 g total ABE is produced from each kg lignocellulose, utilizing about 1 g cellulase per each Gram produced butanol (Tao et al., 2014). Recently, utilization of hemicellulose content of lignocellulose for biobutanol production has been suggested based on the ability of solvent producing *Clostridia* in efficient utilization of hemicellulose derived pentoses (Mirfakhar et al., 2020). In this regard, a number of studies were devoted to thermostable xylanases for biobutanol production (Xin and He, 2013). characterized cellulase-free and thermo-alkali-stable xylanase by isolated anaerobic bacterium (Kluyvera sp. Strain OM3) and utilized it for biobutanol production leading to 1.2 g/L butanol from hemicellulose content of palm oil fiber.

### Pulp and Paper Industry

Xylanase and cellulase that have resistance to high pH and are very stable at elevated temperatures have tremendous potential for application in pulp and paper industries (Cui et al., 2015). Cellulase and xylanase have been reported to improve the dissolved pulp concentration, purity, brightness, and permeability of fiber surfaces, improving paper strength and improving the diffusion of bleaching chemicals (Yang et al., 2019). An experiment with cellulases showed a significant energy reduction (20–40%) in the refinement phase and improved hand-sheet strength (Yang et al., 2019). Highly thermostable endo-xylanase that has maximum activity at a temperature of 60°C and pH of 5.0 was synthesized from *Streptomyces griseorubens* LH-3 this Purified xylanase was utilized in the process of bio bleaching eucalyptus Kraft pulp, and results showed an increase in brightness by 14.5% and decreased kappa numbers by 24.5%. Because it possessed all of these industrially suitable attributes, it could be used in pulp and paper manufacturing as a bio bleaching agent (Wu et al., 2018). Similarly, the bio bleaching effect on rice straw pulp was evaluated using the Thermo-alkali-stable xylanase produced from *Bacillus* tequilensis strain UD-3 isolated from a hot spring. Xylanase. This xylanase shows optimum activity at a temperature of 50°C and pH8.0. There was a remarkable reduction in the various compound such as reducing sugars (50%), phenolic (29.19%), hydrophobic (33.20%), and lignin compounds (35.86 and 40.48%) during the Xyl + Zn treatment of pulp samples when compared to the activity of just xylanase (Patel and Dudhagara, 2020). A study was carried out on enzyme cocktail containing 9.9 IU/g of cellulase and 3811 IU/g of xylanase obtained from *Trichoderma longibrachiatum* MDU 6 for deinking of different types of papers. And it was discovered that it effectively removes ink from old newspapers and significantly remove chromophores, phenolics and hydrophobic compounds (Chutani and Sharma, 2016).

### Food Industries

Cellulases from fungi and bacteria have the potential for application in food production (Singh A. et al., 2021). The juice industry uses cellulases in conjunction with other enzymes to increase productivity and yield, improve extraction methods, clarify and stabilize juices (Chakraborty et al., 2016). They can further lower the viscidity of nectar and puree made from fruits such as apricot, mango, pomegranate, pear, and peach, and they can be used to extract flavonoids from flower nectars and seeds (Uzuner and Cekmecelioglu, 2019). As part of the wine production process, cellulase is combined with other enzymes to improve the yield and quality of the wine. Enhancement maceration, better color development, clarification, and finally improved wine consistency and quality are the main benefits of these enzymes. β-glucosidases can be used through modifications of glycosylated precursors to improve wine aroma (Chakraborty et al., 2016). In addition to higher yields, less heat damage, and shorter processing times, cellulase-mediated extraction is much more preferable to conventional methods. Phenolic compounds can be extracted from grape pomace using cellulase. They are also used to enhance the aroma and taste of citrus fruits as well as reduced their bitterness. Combining β-Glucosidases with pectinase can alter the structure, taste, and aroma of vegetables and fruits (Toushik et al., 2017). Olive oil has so many health benefits; in addition to being a good source of monounsaturated fat, it is also associated with reduced risks of stroke and heart disease, it contains fatty acids, vitamin E, and it also contains some phenolic elements and antioxidants, cellulase can be efficiently utilized when combined with other enzymes to extract olive oil (Bisht et al., 2015).

In bread making, xylanases are used with other enzymes because they have the potential benefit of enhancing the volume of the bread and contributing to its improved quality and they allow faster baking times and increase yields (Tekkol et al., 2017). Wheat flour is treated with xylanase to break down hemicellulose, increasing the binding of water to the flour, allowing it to become softer and more elastic (Dahiya and Singh, 2019). The taste, texture, and palatability of biscuits can also be enhanced using xylanase (Rosmine et al., 2017). In the brewing industry, xylanases are used to hydrolyze the barley cellular wall; using xylanases reduces the viscosity of beer and the cloudy appearance of the beer (Yu et al., 2021).

### Textile Industries

Thermostable cellulase and xylanase are widely used in the textile industries for stone washing of Jeans, Biopolishing of cotton and other fabrics that contains cellulose (Anish et al., 2007; Escuder-Rodríguez et al., 2018). Biopolishing is typically done after desizing, an enzymatic process that involves temperatures over 70°C. Hence, cellulases and xylanases operating at such elevated temperatures could be useful for combining the two enzymatic processes (Bala and Singh, 2017). Cellulase and xylanase are also used in detergent industries they are incorporated with other catalytic agents into detergents; all of these enzymes help to break down the chemical bonds present in dirt, which are more effective at higher temperatures (Rigoldi et al., 2018). Thus, they are highly stable at extreme temperatures 60°C and pH ranges 9.0 to 11.0, and detergent resistant. Numerous thermostable cellulase and xylanase that are detergent tolerant have been studied (Frock and Kelly, 2012; Bagewadi et al., 2016).

### Animal Feed Industries

Thermostable cellulases and xylanases are used in animal feed industry because of their ability to enhance digestibility and quality of animal feed (Bedford, 2018; Costa et al., 2019). A study was carried out on xylanase 50,316 produced from *Pseudomonas* fluorescens, it have the ability to hydrolyze the glycosidic linkages of the xylan found in animals feed (Van Dorn et al., 2018). Similar, a study was conducted on thermostable xylanase as dietary supplement and its effect on the viscosity of digesta as well as live performances of broiler chicks was also evaluated. This enzyme proved to be effective in improving bird performance as well as the dietary in poultry up to 21 days (Barasch and Grimes, 2021). A thermostable carbohydrases AC1 which contains *β*-1,4-glucanase, endo-cellulase and cellobiohydrolases activities was expressed in corn for easy inclusion in animals feed, it was used to feed 5 weeks old pigs and the study show no adverse effect on the performance metrics of the pig and the digestibility of this feed was greatly improved (Lessard et al., 2021).

## Conclusion and Future Perspectives

To turn the vision of environmentally friendly lignocellulosic ethanol technology into a reality, there is a significant role in developing thermostable cellulases and xylanases. Many researchers have isolated and characterized thermostable cellulases and xylanases from thermophilic and hyperthermophilic microbes from different environments. So far, metagenomic libraries have been an excellent source of novel thermostable enzymes. Any industrial enzyme that can react rapidly at high temperatures is most desirable as it reduces the need for enzyme and lowers the rate of microbial contamination, which in turn shortens the time needed for the conversion of lignocellulosic polymers into biofuel and valuable products. Some of the few strategies that have been employed for *in vitro* production of thermostable enzymes with improved catalytic characteristics for application in various industries includes; utilization of mesophilic host for heterologous expression of these proteins, strain enhancement, genetic and metabolic engineering. In order to understand and improve the catalytic activity, thermostability, synergism with other enzymes, and evolutionary relationships of cellulases and xylanases it is very important to biochemically characterized these enzymes systemically. Presently, directed evolution, semi-rational design, structure-guided rational design, nanotechnology and metagenomics library are the most alluring approaches for the synthesis of novel cellulase and xylanase. The genomes of several hyperthermophilic microbes are now available, which could lead to new insights into thermostable cellulases and xylanases. Currently the advancement in metabolic and genetic engineering, molecular microbiology, and structural biochemistry, have helped in synthesis of thermostable cellulases and xylanase. The use of thermostable cellulases and xylanase to transform lignocellulosic biomass into value-added green products can be applied in many sectors, but yet there are many approaches to be explored and developed. Additionally, advancements in bioinformatics will lead to a better understanding and selection of biocatalysts with enhanced properties.
